# Discovery and profiling of small RNAs responsive to stress conditions in the plant pathogen *Pectobacterium atrosepticum*

**DOI:** 10.1186/s12864-016-2376-0

**Published:** 2016-01-12

**Authors:** Stanford Kwenda, Vladimir Gorshkov, Aadi Moolam Ramesh, Sanushka Naidoo, Enrico Rubagotti, Paul R. J. Birch, Lucy N. Moleleki

**Affiliations:** Department of Microbiology and Plant Pathology, Forestry and Agricultural Biotechnology Institute (FABI), University of Pretoria, Pretoria, South Africa; Kazan Institute of Biochemistry and Biophysics, Kazan Scientific Center, Russian Academy of Sciences, Kazan, Russia; Department of Botany and Plant Physiology, Kazan Federal University, Kazan, Russia; Department of Genetics, Forestry and Agricultural Biotechnology (FABI), University of Pretoria, Pretoria, South Africa; Genomics Research Institute, Centre for Microbial Ecology and Genomics (CMEG), University of Pretoria, Pretoria, South Africa; Division of Plant Sciences, College of Life Sciences, University of Dundee (at The James Hutton Institute), Errol Road, Invergowrie, Dundee, DD25DA Scotland UK

**Keywords:** Small RNAs, Strand-specific RNA-seq, Pectobacterium atrosepticum, *in silico* prediction, Transcriptome, Riboswitches, 5′ UTR, 3′ UTR

## Abstract

**Background:**

Small RNAs (sRNAs) have emerged as important regulatory molecules and have been studied in several bacteria. However, to date, there have been no whole-transcriptome studies on sRNAs in any of the Soft Rot *Enterobacteriaceae* (SRE) group of pathogens. Although the main ecological niches for these pathogens are plants, a significant part of their life cycle is undertaken outside their host within adverse soil environment. However, the mechanisms of SRE adaptation to this harsh nutrient-deficient environment are poorly understood.

**Results:**

In the study reported herein, by using strand-specific RNA-seq analysis and *in silico* sRNA predictions, we describe the sRNA pool of *Pectobacterium atrosepticum* and reveal numerous sRNA candidates, including those that are induced during starvation-activated stress responses. Consequently, strand-specific RNA-seq enabled detection of 137 sRNAs and sRNA candidates under starvation conditions; 25 of these sRNAs were predicted for this bacterium *in silico*. Functional annotations were computationally assigned to 68 sRNAs. The expression of sRNAs in *P. atrosepticum* was compared under growth-promoting and starvation conditions: 68 sRNAs were differentially expressed with 47 sRNAs up-regulated under nutrient-deficient conditions. Conservation analysis using BLAST showed that most of the identified sRNAs are conserved within the SRE. Subsequently, we identified 9 novel sRNAs within the *P. atrosepticum* genome.

**Conclusions:**

Since many of the identified sRNAs are starvation-induced, the results of our study suggests that sRNAs play key roles in bacterial adaptive response. Finally, this work provides a basis for future experimental characterization and validation of sRNAs in plant pathogens.

**Electronic supplementary material:**

The online version of this article (doi:10.1186/s12864-016-2376-0) contains supplementary material, which is available to authorized users.

## Background

The importance of small RNAs (sRNAs) in bacterial gene expression regulation is now broadly appreciated [1, 2]. sRNAs play essential regulatory roles in diverse processes including metabolic reactions, stress response, biofilm formation and pathogenesis [3]. They act as either activators or repressors of proteins and mRNAs. The length of most of the bacterial sRNAs ranges between 50 and 300 but can reach up to 500 nucleotides [4]. The best studied bacterial regulatory sRNAs are those that act through base-pairing interactions with target RNAs, usually modulating gene expression post-transcriptionally by controlling the translation and stability of mRNAs. The majority of these are *trans*-acting sRNAs found within intergenic regions (IGRs). *Trans*-acting sRNAs typically regulate mRNAs encoded at different genomic locations on the chromosome in response to changes in environmental conditions [1]. Furthermore, *trans*-encoded sRNAs tend to have limited complementarity with their target RNAs and require the RNA chaperone Hfq to facilitate their pairing with mRNA targets [4]. In contrast, *cis*-encoded antisense RNAs (asRNAs), also referred to as naturally occurring RNAs, are expressed on reverse strands opposite to annotated genes and have extensive complementarity with their target mRNAs [4]. Antisense RNAs are thought to play physiological roles such as repression of genes encoding potentially toxic proteins [5]. Additional roles of asRNAs include blocking the translation of mRNA transcripts encoded on the opposite strand and directing their RNAse III-mediated cleavage [4]. Other important classes of sRNAs include 1) riboswitches (leader sequences), which form part of the mRNA they regulate and usually present in the 5′ UTR regions; 2) sRNAs which interact with proteins and modify their activities by mimicking their RNA or DNA targets, and 3) sRNAs with intrinsic regulatory activities [4].

The advent of RNA-seq for the resolution of messenger and structural RNAs has facilitated the analysis of vast numbers of sRNAs with increased sensitivity [6, 7]. An additional benefit of RNA-seq approaches is that information about the direction of transcription can be resolved using directional RNA-seq (strand-specific RNA-seq; ssRNA-seq). This information is important for the detection of non-coding (nc) RNAs as well as 5′ and 3′ untranslated regions (UTRs), antisense transcripts and determination of overlapping features within the genome [6]. Combining deep sequencing with computational (*in silico*) prediction methods is emerging as an important approach for sRNA detection in bacterial genome sequences [8, 9].

*Pectobacterium atrosepticum* is an important plant pathogen belonging to the bacterial family *Enterobacteriaceae* [10]. This pathogen causes major yield losses globally through blackleg disease on potato plants in the field and potato tuber soft rot diseases during post-harvest storage. Most of the information on pectobacteria concerns their interaction with plant hosts, and little is known about how these bacteria spend much of their life outside of the host [11]. However, it is known that *P. atrosepticum* is able to utilize various adaptive programs that enable bacteria to survive under adverse conditions [12, 13]. In a previous study, we showed that realization of these programs under nutrient-deficient conditions (starvation) is coupled with an increased transcript abundance of stress responsive genes in *P. atrosepticum,* and bacterial cells undergo morphological and ultrastructural changes [14]. In the current study we have evaluated the possible participation of sRNAs in bacterial starvation-induced stress response.

Few experimental studies on sRNAs have been carried out in *P. atrosepticum*. A well-known regulatory sRNA in *P. atrosepticum* is *rsmB*. This sRNA binds the RsmA protein, which is a homologue of *Escherichia coli* CrsA, a carbon storage regulator, and modulates its activity. In *P. atrosepticum* the RsmA/*rsmB* system regulates the production of virulence factors [15–17]. Moreover, a regulatory RNA antisense to the *expI* gene transcript, which encodes the synthase of mediators of quorum sensing (acyl-homoserine lactones), was found recently in *P. atrosepticum* [18].

In the present study, identification of sRNAs in the complete genome of *P. atrosepticum* SCRI1043 was undertaken using *in silico* prediction and experimental validation via strand-specific RNA-sequencing. Both true (and/or known) and potentially novel sRNA candidates expressed under starvation conditions were identified. Differential expression analysis indicated that many of these sRNAs increase in abundance during exposure of bacteria to starvation compared to rich medium conditions, suggesting an important role of sRNAs in the survival of *P. atrosepticum* cells during nutrient deficiency induced stress.

## Results and discussion

### Strand-specific RNA-seq detection of *P. atrosepticum* sRNAs under starvation-conditions

**Fig. 1:**
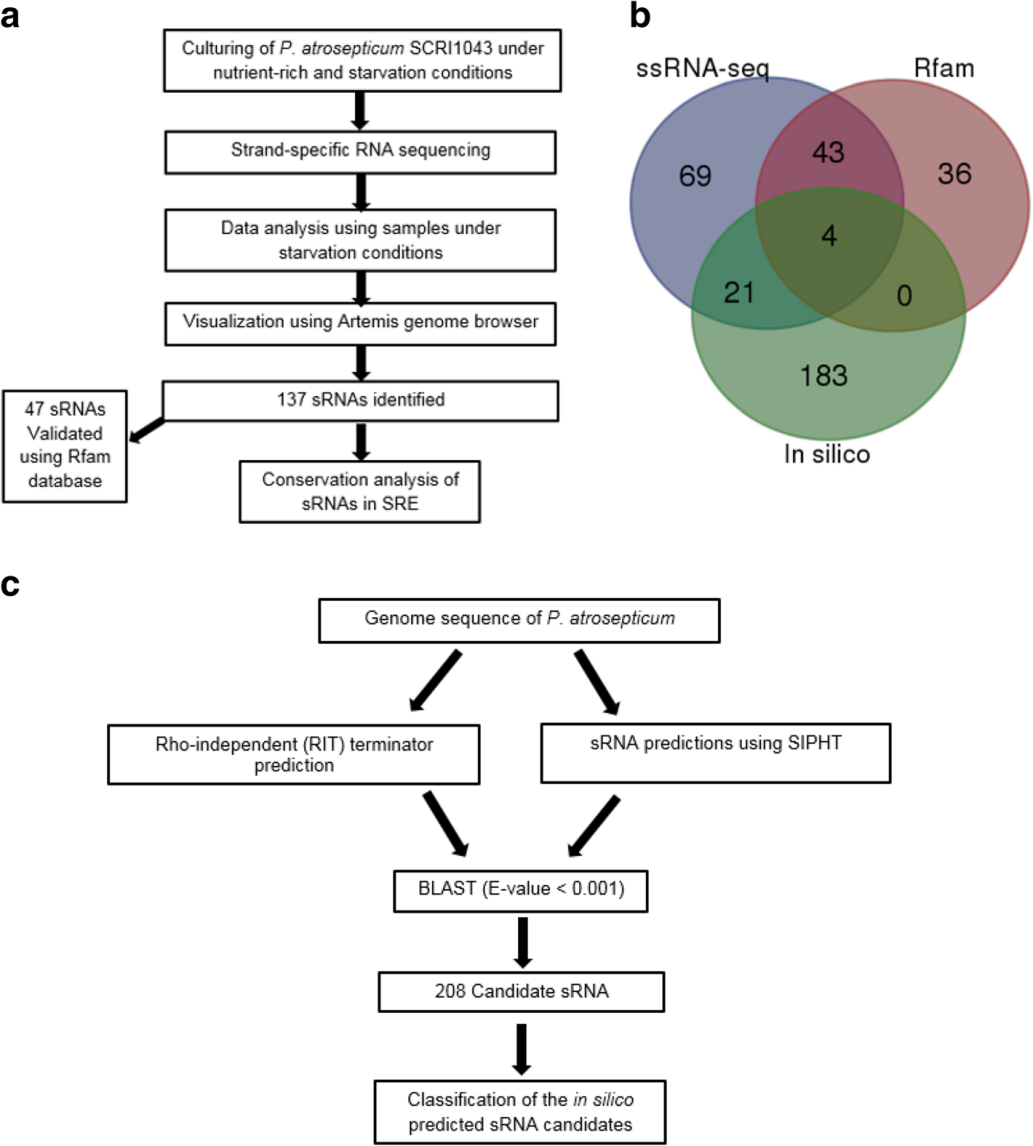
Scheme for sRNA identification. **a** Determination of sRNA using strand-specific RNA seq of *P. atrosepticum* cultured under starvation conditions. **b** Comparison of sRNAs identified by strand-specific RNA-seq with sRNA candidates predicted for *P. atrosepticum* in Rfam database and sRNAs predicted computationally in this study. **c** Computational (*in silico*) sRNA prediction

**Fig. 2:**
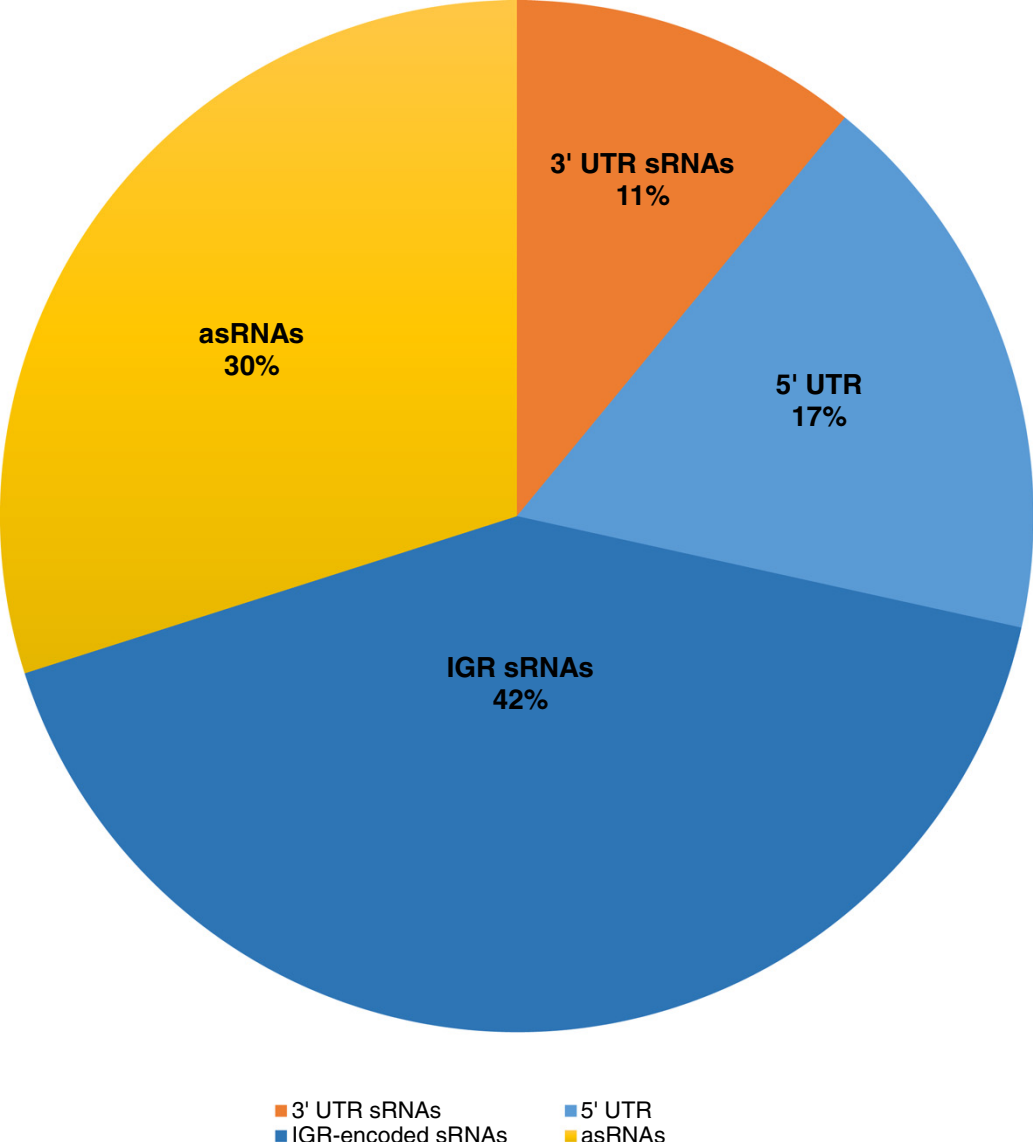
Classification of sRNAs identified using ssRNA-seq into five classes: These include; IGR/ *trans*-encoded sRNAs, asRNA, 5′UTR (riboswitches), 3′ UTR and sense sRNAs (seRNAs), based on their proximity and location with regards to CDS regions

For experimental detection of sRNAs in *P. atrosepticum* SCRI1043, we used a combination of *in silico* and directional whole-transcriptome cDNA sequencing (strand-specific RNA-seq) (Fig. 1). The experimental approach for determination of sRNA in *P. atrosepticum* is outlined in Fig. 1a. A total of 27.4 and 26.1 million paired-end (PE) reads were obtained from nutrient rich and starvation conditions, respectively. By using SAMtools [19], PE reads mapped to each strand were extracted. Thus, enabling visualization of the sequence (PE) read alignments on the genome in a strand-specific manner. Visual inspections enabled the identification of candidate sRNA transcripts by manually analysing the position of PE reads with respect to annotated protein-coding regions (CDS). This can be a particularly powerful approach to identify sRNAs and resolve their genomic positions because reads that map to intergenic regions may represent previously unannotated transcriptionally active non-coding sRNAs [20]. Only sRNA candidates with a length between 50 to 500 nucleotides were considered to be true positive sRNAs candidates. This technique enabled identification of a total of 137 sRNA candidates expressed under starvation condition (Additional file 1: Table S1). These candidate sRNAs were classified into four distinct sRNA groups based on their position in relation to adjacent CDSs: IGR/ trans-encoded sRNAs, asRNA, 5′ UTR (riboswitches), and 3′ UTR sRNAs (Fig. 2). An *in silico* approach (described in the section below) was employed to determine the putative transcriptional start sites (TSS) of the identified 137 sRNAs and to resolve their 5′ ends. Only predicted TSS with transcription factor binding sites were considered as bona fide promoters. Thus, using this filter, TSS were identified upstream of 118 sRNA genes (Additional file 2: Table S2).

### Identification of 3′ UTR encoded sRNAs

We identified 15 sRNAs encoded within the 3′ UTR regions of mRNA (referred to in this study as 3′ UTR sRNAs) (Fig. 2). It is now appreciated that sRNAs not only originate from intergenic regions as independent transcripts but are also transcribed from 3′ regions of coding mRNA [21]. These 3′ UTR sRNAs are generated either by means of mRNA transcript processing or as primary transcripts from an internal promoter within the mRNA coding sequence as in the case of *dapZ* sRNA [22]. Thus, based on how they are produced, 3′ UTR encoded sRNAs can be divided into 2 groups, that are: 1) sRNAs transcribed from an independent promoter located inside the overlapping mRNA gene or 3′ UTR region (Type 1); and 2) sRNAs which are originated from the processing of the parent mRNA (Type 2) [23]. Hence we used our ssRNA-seq data to determine whether the identified 3′ UTR embedded sRNAs are transcribed independently from the parent mRNA. Ten 3′ UTR sRNAs were considered to be independently transcribed based on comparisons of sRNA and parent mRNA RPKM (reads per kilobase of transcript per million mapped reads) values and the presence or absence of an internal promoter (Table 1). To determine the putative 5′ ends and fundamental types of the detected 3′ UTR sRNAs based on their biogenesis, we extracted each sRNA sequence plus 200 nt upstream of the start position of each sRNA and performed promoter predictions using BPROM program (http://www.softberry.com/berry.phtml?topic=bprom&group=programs&subgroup=gfindb). This approach led to the identification of 14 distinct putative promoter sites (transcriptional start sites; TSS) embedded within the coding or 3′ UTR regions of the parent mRNA upstream of each 3′ UTR sRNA gene (Table 1). In addition, transcription factor binding sites were also detected within the predicted promoter regions. Taken together, the presence of putative internal promoter sites upstream of sRNAs TSS and the predicted transcriptional factor binding sites for each promoter, strongly suggests that fourteen 3′ UTR sRNAs are type 1. Nine of which were also differentially expressed compared to their parent mRNAs based on RPKM values, further indicating evidence of independent expression. The remaining sRNA reg_seq13 could be a product of mRNA processing, thus type 2 since no internal promoters supported by transcription binding sites were predicted for this sRNA. Overall, since the sRNA 5′ ends and subsequent TSSs were predicted computationally, we were not able to determine whether these sRNAs possessed the characteristic 5′-triphosphate (5′-PPP) cap common to type 1 sRNAs in this present study.

**Table 1:** 3′ UTR encoded sRNAs

		sRNA			RPKMs		Expression	Predicted	sRNA promoter and start site			Transcription Factor
sRNA name	Parent mRNA	Start	end	length	sRNA	mRNA	(based on RPKMs)	promoters	−35	−10	TSS	binding site
fwd_4	*rbsB*	14355	14537	183	596.8	102.8	Independent	1	14317	14342	14357	rpoD17, cynR, rpoD15, rpoD16, phoB
fwd_6	*polA*	28634	28755	122	549.8	110.5	Independent	1	28656	28676	28691	cytR, arcA, crp, rpoD15, rpoD17
fwd_15	*ECA0044*	55039	55291	253	66.8	27.4	Independent	1	55009	55029	55044	rpoD17, fis, rpoD15, rpoD16, phoB
fwd_19	*expI*	126355	126501	147	338.5	588.1	Co-expression	1	126418	126435	126450	metR, rpoD16
reg_seq13	*aldA*	139913	140154	242	136.8	30.4	Independent	1	140046	140063	140078	
reg_seq27	*ECA0332*	380584	380826	243	1660.5	389.3	Independent	1	380610	380631	380646	metJ
reg_seq31	*ECA0449*	515673	515910	238	363.3	528.7	Co-expression	1	515790	515813	515828	glpR, ihf, argR2, nagC, argR2, fnr, fis
reg_seq43	*mdH*	758603	758857	255	1759.1	1895.7	Co-expression	2	758751	758770	758789	glpR, fis, arcA, purR
									758444	758464	758479	
reg_seq142	*ECA2516*	2832294	2832530	237	514.2	77.5	Independent	1	2832185	2832205	2832220	purr, rpoD16
fwd_rfam4	*rpiL*	255260	255321	62	775.5	1856.1	Independent					lrp, hns
comp_seq5	*glnA*	34992	35234	243	858.8	1209.3	Co-expression	1	35262	35241	35226	rpoD15, rpoD16, phoB
comp_seq11	*slmA*	164026	164334	309	196.4	454.0	Independent	1	164395	164375	164360	arcA, rpoD17, rpoD15, rpoD16, phoB
comp_seq130	*ECA2950*	3295111	3295347	237	197.9	222.9	Co-expression	2	3295196	3295176	3295161	rpoD16, argR, arcA, ihf
									3295506	3295485	3295470	
rev_rfam22	*glpC*	4651380	4651485	109	348.2	40.8	Independent	1	4651583	4651562	4651547	Ada, rpoE, tyrR, fur, fur
dapZ	*ECA3872 (dapB)*	4332178	4332288	111	20.9	110.8	Independent	1	4332302	4332284	4332269	Ihf, argR2, rpoD16, argR,, fis, crp

The 137 sRNAs identified using strand-specific RNA-seq approach were checked against known *P. atrosepticum* SCRI1043 non-coding RNA descriptions on the Rfam database [24]. For this analysis, all descriptions for tRNAs, rRNAs and CRISPR RNAs were excluded. This also served to assess the efficiency of the strand-specific RNA-seq method in detecting sRNA transcripts. In total 56.6 % (47/83) of the known *P. atrosepticum* sRNAs in the Rfam database were identified using ssRNA-seq of cells cultured under starvation conditions (Fig. 1b and Additional file 1: Table S1).

### Computational prediction of sRNA in the *Pectobacterium atrosepticum* genome

Even though ssRNA-seq is a powerful tool for identification of sRNAs, it might be subject to some limitations. For example, since the formation of particular sRNAs is highly dependent on culture conditions, it is not possible to unravel the whole pool of sRNAs that is encoded in the genome of the target microorganism within the frameworks of a given experiment. Consequently, a combination of experimental and computational identification of sRNA is often seen as a more comprehensive approach towards identification of sRNAs [25, 26]. Hence, in addition to ssRNA-seq, an *in silico* sRNA analysis was performed according to computational methods implemented previously [27], with some modifications (see Fig. 1c for a schematic representation of the computational prediction strategy).

An initial step towards *in silico* sRNA candidate disclosure consisted of identification of predicted rho-independent terminators (RITs) in the *P. atrosepticum* SCRI1043 genome. Since about 72 % of known sRNAs located within IGRs possess a RIT, computational methods based on prediction of RIT signature sequences have emerged as valuable algorithms for the detection of sRNA molecules [8, 27]. In intergenic and antisense to annotated open reading frames (ORF) in the *P. atrosepticum* SCRI1043 genome we detected a total of 1598 putative terminators (including both canonical and non-canonical terminators’ candidates) with the ‘Greatest ΔG’ i.e. the most negative ΔG (free Gibbs energy) value. From the 1598 putative sRNA identified, 1165 were filtered out and excluded from further analysis due to the fact that their RITs were located less than 60 nucleotides downstream from stop codons of preceding annotated ORFs within the same strand. This resulted in identification of 433 sRNA candidates of 226–248 nt in length (Additional file 3: Table S3). To be more confident about the accuracy of the rho-independent terminator based prediction strategy used, a second prediction tool (SIPHT) [28] was employed. Herewith, the filtered set of sRNA candidate signatures was compared against sRNA predictions for *P. atrosepticum* SCRI1043 from the SIPHT web interface by means of BLAST local pairwise alignments using the genomic similarity search tool YASS [29], with standard parameters. Each comparison was made on both regular and complementary strands separately. As a result, a total of 105 and 101 matches (E-value < 0.001) were identified, partially or fully overlapping, for the forward and complementary strands, respectively. This additional filtering step combining comparative genomics with RIT based predictions yielded 206 sRNA candidates in *P. atrosepticum* SCRI1043 (Additional file 4: Table S4). Similarly to sRNA detected using ssRNA-Seq, predicted sRNAs were further classified into five distinct sRNA groups based on their position in relation to adjacent CDSs (results not included).

### Comparison of RNA-seq results with computational sRNA predictions

The 208 candidate sRNAs identified computationally were compared to the 137 sRNA transcripts identified using ssRNA-seq. Only 25 of the *in silico* predicted sRNA candidates were also identified by RNA sequencing (Table 2). Such an incomplete overlap between computational sRNA predictions and deep sequencing detection has been noted in previous studies [2, 8, 9, 30]. It is possible that the discrepancy observed here could be largely because experimental detection of sRNAs was restricted to sRNAs expressed under one condition, viz starvation. Hence, it may well be that increasing the number of conditions in which RNA is harvested could lead to bridging the gap between *in silico* predicted and ssRNAseq identified sRNAs. Lastly, the disparity could be due to the presence of false positive *in silico* predictions as well as the elimination of sRNAs associated with RITs in close proximity to CDS regions when using RIT identification based *in silico* predictions. Nonetheless, the lengths of the majority of the *in silico* predicted sRNA transcripts were comparable to the sizes deduced from the strand-specific RNA-seq sRNA detections for the confirmed sRNA candidates.

**Table 2:** *In silico* predicted sRNA candidates confirmed by strand-specific RNA-seq

sRNA candidate	sRNA class	sRNA type	sRNA start	sRNA end	sRNA length
reg_seq3	3′UTR: *polA*		28634	28755	122
reg_seq3b	IGR	spot42 sRNA	28756	28882	127
reg_seq13	3′ UTR: *aldA*		139913	140154	242
reg_seq27	3′ UTR: *ECA0332*	TPP riboswitch	380584	380826	243
reg_seq27b	5′ UTR: *icc*	isrH (Hfq binding sRNA)	380825	380917	93
reg_seq31	3′ UTR: *ECA0449*		515673	515910	238
reg_seq34	3′ UTR: *topB*	STAXI sRNA	601635	601862	228
reg_seq34b	antisense: *ECA0527*	STAXI sRNA	601860	602087	228
reg_seq43	3′ UTR: *mdh*	Glycine riboswitch	758603	758857	255
reg_seq67	IGR		1185902	1186147	246
reg_seq70	antisense: *ECA1096*		1225748	1226182	435
reg_seq76	5′ UTR: *mend*	TPP riboswitch	1379050	1379349	300
reg_seq109	antisense: *osmB*	TPP/ isrH	2217586	2217946	361
reg_seq129	IGR	Trp leader	2602697	2602931	235
reg_seq133	5′ UTR: *ansA*	RtT and TPP	2651356	2651598	243
reg_seq142	3′ UTR: *ECA2516*		2832294	2832530	237
comp_seq5	3′ UTR: *glnL*	TPP/ isrH	34992	35234	243
comp_seq11	3′ UTR: *slmA*	isrH	164026	164334	309
comp_seq16	5′UTR: *ECA0353*	TPP	403257	403655	399
comp_seq49	IGR	TPP	1218529	1218729	201
comp_seq55	5′ UTR: *ECA1196*	TPP/ isrH	1358123	1358268	146
comp_seq111	IGR	TPP/ isrH	2881355	2881546	192
comp_seq130	3′ UTR: *ECA2950*	TPP and RtT	3295111	3295347	237
comp_seq204	5′ UTR: *sotB*		4828158	4828349	192
comp_seq217	5′ UTR: *ECA4506*		5046219	5046427	209

### Functional annotation of RNA-seq detected sRNAs

To describe and assign biological functions to the 137 sRNAs detected by strand-specific RNA-seq (including those confirmed by *in silico* predictions), we used the Rfam database (version 11.0) [31] and the RNAspace platform [32]. The RNAspace platform comprises a suite of ncRNA prediction tools. Similarity searches on the RNAspace platform were restricted to comparative analysis and homology searches using BLAST/ YASS (sequence homology tools) against the Rfam 10.0 seed database and three RNA motif search tools, DARN, ERPIN and INFERNAL. In total, 68 sRNAs representing true (and/or known), previously described sRNA sequences were assigned into 6 functional classes (E-value < 0.001), and these included: 1 ribozyme, 21 riboswitches (consisting of 6 types), 14 RNA elements (10 different types), 30 sRNAs (including 9 Hfq-binding sRNAs), 1 asRNA and 1 tmRNA (Table 3). Amongst these, we characterized 13 sRNA sequences which were previously uncharacterized within the *P. atrosepticum* genome by means of Blast (e-value < 0.001) and secondary structure predictions using the RNAfold Webserver [33]. No functional classes were assigned to the remaining 69 sRNAs computationally, suggesting that they could be potentially novel sRNA candidates in *P. atrosepticum*.

**Table 3:** Functional annotation of the 68 true (and/or known) sRNAs identified by strand-specific RNA-seq

sRNA name	sRNA type	Characterized in this study (RNAspace)	Previously characterized (Rfam)	Total
RNaseP	Ribozyme		1	1
tmRNA			1	1
*Cis*-regulators				
	Riboswitches			
TPP riboswitch		6	3	9
TPP or isrH		8		8
glycine riboswitch		2	2	4
FMN			1	1
lysine riboswitch		1		1
MOCO_RNA_motif			1	1
yybP-ykoY			1	1
	RNA elements			
alpha_RBS			1	1
cspA			2	2
greA			1	1
his_leader			1	1
JUMPStart			2	2
leucine operon leader			1	1
P26			1	1
rne5			1	1
RtT		1	2	3
trp_leader			1	1
*trans*-encoded sRNA				
	sRNA			
*STAXI*			3	3
6S			1	1
*crcB*			1	1
*csrB*			1	1
*glmY* (*tke1*)			1	1
*Rye*		1		1
*sraC* (*ryeA*)			1	1
*STnc240*			1	1
*StyR-44*			5	5
*t44*			1	1
	Hfq binding sRNA			
*frnS*			1	1
*isrH*		2		2
*glmZ* (*sraJ*)			1	1
*omrA*			1	1
*rprA*			1	1
*ryhB*			1	1
*ryeB (sdsR)*			1	1
*sgrS*			1	1
*spot 42*			1	1
antisenseRNA (asRNA)	asRNA			
*HPnc0260*			1	1
Total		21	47	68

Most of the detected riboswitches in this study corresponded to thiamine pyrophosphate (TPP) (Thi-box) riboswitches (Table 3). Bacterial riboswitches are embedded within the leader sequences (5′ UTR regions) of numerous metabolic genes and act by repressing or activating their cognate genes at the translational level in gram-negative bacteria [34]. Most thiamin-regulated genes encode transporters in different bacterial organisms [35]. For example, TPP riboswitches identified are present upstream of genes involved in potassium transport (*trkD*), amino acid biosynthesis (*argG*), and genes related to the biosynthesis of secondary metabolites (*menD*). Generally, TPP riboswitches are found upstream (5′ UTR regions) of many genes key in metabolic processes which use TPP as a cofactor [35]. In this study, we also detected other riboswitches other than TPP-type riboswitches, that include Flavin mononucleotide (FMN), glycine, lysine, yybP-ykoy and MOCO RNA motif riboswitches.

Some of the detected RNA elements (leader sequences) were located upstream of operons or genes involved in biosynthesis of amino acids including leucine, histidine and tryptophan biosynthesis; and polysaccharide synthesis (Additional file 1: Table S1). It therefore seems plausible that most of the detected *cis*-regulatory elements are engaged in regulating processes involving substrate transport and biosynthesis in *P. atrosepticum*.

### Conservation analysis of predicted sRNAs

**Fig. 3:**
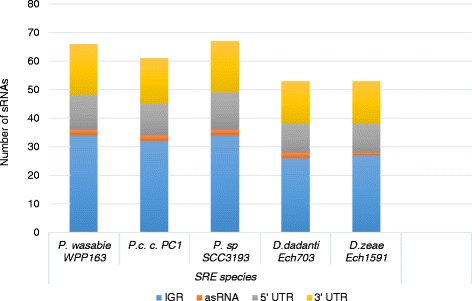
Summary of sRNAs conserved in soft rot *Enterobacterieace*

The vast majority of known sRNAs are typically highly conserved across genera [36]. We therefore analysed the conservation of identified sRNAs in *P. atrosepticum* SCRI1043 in five soft rot *Enterobacteriaceae* species whose complete genome sequences are available on GenBank. The 68 true/ known sRNA sequences with assigned functional classes were used for the conservation analysis. BLASTn analysis (E-value < 0.0001) using the YASS tool against *P. carotovorum* subsp*. carotovorum* PC1, *P. wasabiae* WPP163, *Pectobacterium spp.* SCC3193, *Dickeya dadantii* Ech703 and *D. zeae* Ech1591 complete genome sequences revealed that most sRNAs are conserved within the soft rot bacterial species with 42 sRNAs (including 13 *trans*-encoded sRNAs, 18 riboswitches, 10 RNA elements, and 1 asRNAs) being present in all five SRE species (Fig. 3 and Additional file 5: Table S5). The high conservation of sRNAs within the SRE species emphasizes their regulatory importance in these bacteria. Six IGR sRNAs were conserved only in the *Pectobacterium* genus and belonged to two RNA families; namely *styR-44*, and *crcB* RNA motif (fluoride riboswitch) sensing fluoride ions and regulating the *crcB* gene (hypothetical protein) which possibly encodes a protein that functions by removing excess fluoride ions from the cell.

**Fig. 4:**
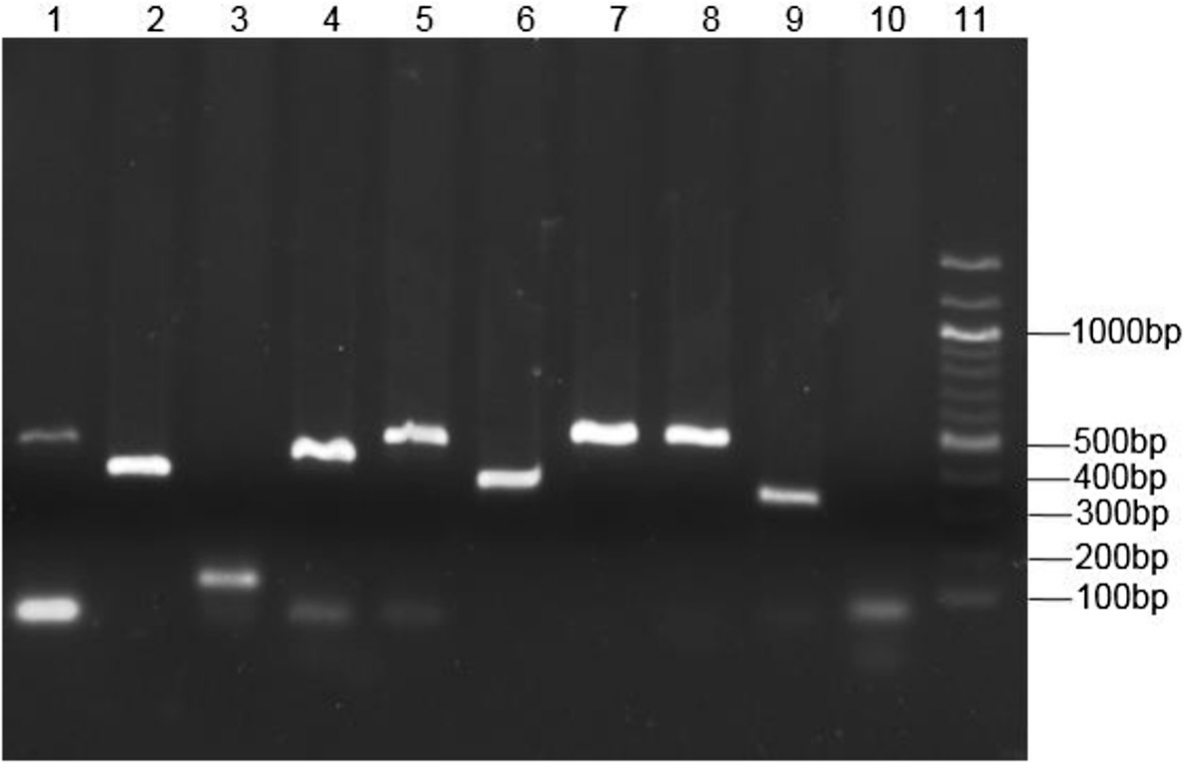
Validation of novel sRNA expression by RT-PCR: Agarose gel electrophoresis of the PCR amplicon fragments of the 9 novel sRNAs. Lane 1. rev_41, Lane 2. rev_13, Lane 3. fwd_6, Lane 4. rev_11, Lane 5. fwd_72, Lane 6. fwd_44, Lane 7. fwd_42, Lane 8. rev_24, Lane 9. rev_39, Lane 10. No reverse transcriptase control, Lane 11. 100 bp DNA Ladder

To be more confident with the 69 potentially novel sRNA candidates detected by ssRNA-seq, we filtered and screened them by checking their conservation within the five representative SRE strains using sequence similarity analysis. Nine of these candidate sRNAs had high sequence conservation (100 % identity and coverage) within SRE strains and only single hits from the BLAST analysis and therefore were considered as novel sRNAs (Table 4). To validate the expression and lengths of the nine novel sRNAs, reverse transcription PCR (RT-PCR) was performed on cDNA of bacteria cells cultured under starvation conditions (Fig. 4). For each of the cDNA samples, a single amplicon that corresponded to the sRNA transcript size identified by ssRNA-seq was observed. As an additional validation step, the nucleotide bases of observed amplicons were confirmed by sequencing and alignment to respective sRNA sequences (Additional file 6: Table S6).

**Table 4:** Novel sRNA candidates obtained using conservation analysis

sRNA Name	Strand	Length	sRNA Class
rev_11	-	420	asRNA: ECA0328
rev_13	-	354	asRNA: ECA0388
rev_24	-	489	asRNA: *rcsC*
rev_39	-	300	5′ UTR: *ilvG*
rev_41	-	480	IGR/ 5′ UTR: *bcsB*
fwd_6	+	122	3′ UTR: *ECA3097*
fwd_42	+	480	5′ UTR: *zipA*
fwd_44	+	336	asRNA: *ECA0910*
fwd_72	+	426	asRNA: *gudP*

### Differential expression of sRNAs under nutrient-rich and starvation conditions

**Fig. 5:**
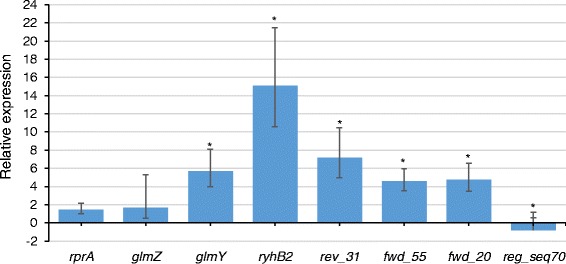
RT-qPCR validation of RNA-seq expression analysis. Relative expression changes of sRNAs were determined using the 2-ΔΔct method by comparing expression in starvation conditions to nutrient-rich. Error bars indicate the standard error of three independent biological replicates. Asterisks represent significant difference at *p <* 0.05 (Students *t*-test)

Application of strand-specific RNA-seq to study the transcriptome of *P. atrosepticum* uncovered an abundance of sRNAs including antisense transcripts, intergenic sRNAs and *cis*-encoded regulatory elements. The number of RNA-seq reads mapping to individual sRNA sequences provides a realistic assessment of relative transcript abundance [37], thus enabling quantification of differential expression of the sRNA transcripts in *P. atrosepticum* cells existing under nutrient-rich and nutrient-deficient (starvation) conditions. The differential expression of sRNAs when growth conditions are changed could suggest potential functions and clarify conditions that induce or repress formation of specific sRNAs [38]. Hence, in order to understand the expression profiles of sRNAs in response to carbon and phosphorus starvation, we compared expression patterns of *P. atrosepticum* cells under nutrient-rich and nutrient-deficient (starvation) conditions. Based on the combined statistics of edgeR package [39] (dispersion = 0.04; q-value < 0.1), and Gfold algorithm (v.1.1.4) [40], which uses a posterior distribution of log fold change for determining expression changes in experiments with single biological replication, thus, overcoming the shortcomings of relying on statistics based on p-value when biological replication is lacking [40]. Subsequently, only sRNAs with significant differential expression from edgeR and Gfold analyses were considered. Thus, a total of 68 sRNA candidates were differentially expressed (Additional file 7: Table S7). Of these, 47 sRNAs were up-regulated under nutrient-deficient conditions (Additional file 7: Table S7) suggesting that they are likely involved in regulatory mechanisms of stress response or adaptation in *P. atrosepticum*. To validate expression profiles identified by ssRNA-seq, we performed reverse transcription quantitative PCR (RT-qPCR) using three biological replicates, on eight selected sRNAs that were differentially expressed under nutrient-rich and starvation conditions. The RT-qPCR results confirmed expression patterns of these eight sRNA transcripts and validated our RNA-seq data (Fig. 5). Selected examples are discussed below.

We noticed that *rprA* was up-regulated (~1.5-fold) in *P. atrosepticum* under carbon-starvation conditions (Fig. 5 and Additional file 7: Table S7). The sRNA *rprA* acts by increasing (positively regulating) the translation of *rpoS* gene transcript [41, 42]. RpoS is a sigma factor that controls the expression of stress responsive genes in bacteria during adverse conditions and stationary phase. We observed that the expression of *rpoS* gene in *P. atrosepticum* was higher during starvation than under nutrient-rich conditions (data not shown). This observation is consistent with previous data demonstrating that RpoS is a principal regulator of the general stress response in bacteria allowing cells to survive environmental challenges as well as prepare for subsequent stresses [43]. This is also consistent with our previous observations, demonstrating that *rpoS* gene expression increases significantly in *P. atrosepticum* under stress conditions [14]. Generally, the regulation of *rpoS* gene expression is known to be modulated at the translational level by at least four sRNA, namely, *arcZ*, *dsrA*, *rprA*, and *oxyS* in response to temperature, osmotic shock, oxidative stress and nutrient deprivation in *E. coli* [41, 44]. Hence, increased *rprA* expression observed in our study in *P. atrosepticum* under nutrient starvation conditions is likely to promote the enhanced translation of *rpoS* mRNA during adaptation of bacteria to starvation conditions.

**Fig. 6:**
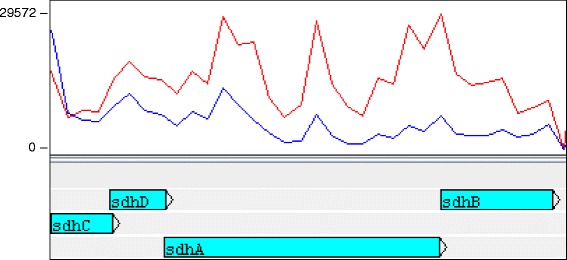
The expression of the *sdhCDAB* operon is relatively lower under starvation compared to growth promoting conditions. Reads mapped from the nutrient-rich condition are represented by the red line. The blue line represents mapped reads from the starvation conditions. Annotated features are labelled below the plot in blue blocks. The y-axis shows the read coverage per coding region (CDS)

*ryhB2*, a 106 nucleotide paralogue of *ryhB* sRNA, was up-regulated by a 15-fold magnitude in *P. atrosepticum* under nutrient-starvation conditions (Fig. 5). Generally, *ryhB,* regulates iron metabolism, including its acquisition and assimilation. *ryhB* acts by down-regulating expression of genes encoding iron-storage and iron-using proteins when iron is in limited supply. The main target genes for *ryhB* include the *sdhCDAB* operon encoding succinate dehydrogenase and *sodB* which encodes the iron-dependent superoxide dismutase [45]. *ryhB* expression level is usually inversely correlated with expression levels of the mRNA for the *sdhCDAB* operon [46]. This is consistent with our observations for *P. atrosepticum*: the transcription of the *sdhCDAB* operon was reduced under starvation conditions compared to the growth-promoting ones (Fig. 6).

Starvation conditions also induced the expression of *glmZ* (~2-fold increase) and *glmY* (5-fold increase) sRNAs in *P. atrosepticum* (Fig. 5). In enteric bacteria, these two sRNAs regulate amino sugar metabolism by activating the expression of *glmUS* operon which encodes the glucosamine-6-phosphate synthase, an essential enzyme in amino sugar metabolism [47]. The regulation by these two sRNAs modulates the transitions between carbon storage and carbon metabolism [48]. The level of *glmY* is increased in the absence of glucosamine-6-phosphate leading to stabilization of *glmZ*. The latter, in turn, activates *glmS* gene expression in an anti-antisense mechanism [48]. GlmS enables cells to utilize the intermediates of glycolytic pathway including the fructose-6-phosphate for production of amino sugars. The glucosamine-6-phosphate is an essential precursor for biosynthesis of essential components of the cell envelope such as peptidoglycan and lipopolysaccharide in gram-negative bacteria. Thus, induction of *glmY* and *glmZ* expression in *P. atrosepticum* under starvation conditions likely indicates the important role of the amino sugar metabolism in adaptive response on this bacterium.

In summary, we have shown that several sRNAs are induced under nutrient-deficient compared to nutrient-rich conditions. We have also shown that induction of these sRNA leads to induction of various genes that potentially play a role in the survival of *P. atrosepticum*. In other members of the *Enterobacteriaceae* family including *E. coli* and *Salmonella*, sRNAs have also been shown to play an important role in adaptation to nutrient limited condition [49]. In these bacteria, sRNAs provide a signal that triggers production of extracellular polysaccharides (EPS) which in turn are involved in biofilm formation [50]. Although *P. atrosepticum* does not readily form biofilms in vitro, the overexpression of a diguanylate cyclase (PleD*), induced formation of biofilms suggesting that biofilm formation in this pathogen is cryptic and can be activated under optimum conditions [51]. Part of the pathogenesis of *P. atrosepticum* is in xylem tissue (when causing black leg disease of potato stems). The xylem is typified by limited nutrients and as such a harsh environment that requires well defined methods of survival. Hence, it is not surprising that many xylem dwelling phytopathogens such as *Xanthomonas*, *Clavibacter*, *Ralstonia* and *Xylella* form biofilms in xylem tissues of their respective hosts. Thus, it is possible that sRNA are extensively involved in the adaptation of *P. atrosepticum* and survival in stem vasculature. Identification of this suite of sRNA will allow us to study the role that these play in survival of this phytopathogen during stem colonisation.

## Conclusions

In conclusion, in this study we have used a combination of strand-specific RNA-sequencing and *in silico* approaches to detect and analyse sRNAs in *P. atrosepticum* SCRI1043. We demonstrated the efficiency of ssRNA-seq in detecting sRNAs and determining the sRNA expression levels in response to specific bacterial growth conditions. A total of 137 sRNAs and sRNA candidates were experimentally detected in this study. We successfully determined sRNAs (that are riboswitches, *trans*-encoded sRNAs, 3′ UTR sRNAs and asRNAs) that may play key roles in regulating stress responses. Most of the identified sRNAs in *P. atrosepticum* are conserved within the soft rot enterobacteria (SRE) species suggesting their importance in physiological responses for the SRE species. To our knowledge, this study constitutes the first genome/ transcriptome-wide analysis aimed at the discovery of sRNAs responsive to nutrient-deficiency (starvation) in bacteria. A significant fraction of the unravelled sRNAs appeared to be starvation responsive indicative of their importance in adaptation of bacteria to stress conditions. Determining the biological roles of these sRNAs will broaden our understanding of the diverse regulatory mechanisms they provide in modulating gene expression in *P. atrosepticum* and other SRE species during adaptation to changing environments.

## Methods

### Strains, culture conditions and strand-specific RNA-seq

#### Bacterial strains, media and culture conditions

A strain of *P. atrosepticum* SCRI1043 [10], was used in this study. sRNA profile was analysed in bacterial cells existing under growth-promoting and starvation conditions. The cultures with inoculation titer of 2–3 × 10^6^ CFU (colony forming units) per ml were grown in Luria-Bertani medium [52], with aeration (200 r.p.m.) at 28 °C for 16 h (growth-promoting conditions). Aliquots of these cultures were used for total RNA extraction. The remaining cells were transferred (after double wash) to carbon and phosphorus deficient AB medium containing 1 g l^−1^ NH_4_Cl; 0.62 g l^−1^ MgSO4 × 7H_2_O; 0.15 g l^−1^ KCl; 0.013 g l^−1^ CaCl_2_ × 2H_2_O and 0.005 g l^−1^ FeSO_4_ × 7H_2_O, pH 7.5 and incubated under starvation conditions with initial cell density of 5.4 × 10^8^ ± 6.1 × 10^7^ CFU per ml in glass vials without aeration at 28 °C [53]. Total RNA was extracted from 24 h starving cells.

#### Total RNA preparation

Total RNA was isolated from bacterial cells using the RNeasy Protect Bacteria Mini Kit (Qiagen, USA), according to the manufacturer’s instructions. Contaminating DNA was removed from the samples by DNAse (Qiagen) treatment. RNA was quantified using a Qubit fluorometer (Invitrogen, USA).

### cDNA library construction and bacteria strand-specific RNA sequencing

Library construction and strand-specific sequencing were carried out at the Beijing Genomics Institute (BGI-Shenzhen, China; http://www.genomics.cn/en/index), following the manufacturer’s protocols. Briefly, the rRNA was depleted from 1 microgram of total RNA using the Ribo-Zero Magnetic Gold Kit (Epicenter). TruSeq RNA Sample Prep Kit v2 (Illumina) was used for library construction. RNA was fragmented into small pieces using Elute Prime Fragment Mix. First-strand cDNA was synthesized with First Strand Master Mix and Super Script II (Invitrogen) reverse transcription (25 °C for 10 min; 42 °C for 50 min; 70 °C for 15 min). After product purification (Agencourt RNAClean XP Beads, AGENCOURT) the second-strand cDNA library was synthesized using Second Strand Master Mix and dATP, dGTP, dCTP, dUTP mix (1 h at 16 °C). Purified fragmented cDNA was end repaired (30 min at 30 °C) and purified with AMPureXP Beads (AGENCOURT). Addition of the poly (A) tail was done with A-tailing Mix (30 min at 37 °C) prior to ligating sequencing adapters (10 min at 30 °C). The second-strand cDNA was degraded using the Uracil-N-Glycosylase (UNG) enzyme (10 min at 37 °C) and the product purified by AMPureXP Beads (AGENCOURT). Several rounds of PCR amplification with PCR Primer Cocktail were performed to enrich the cDNA fragments and the PCR products were purified with AMPureXP Beads (AGENCOURT). Sequencing was performed using the Illumina HiSeq™ 2000 platform with pair-end 90 base reads.

### Sequence read processing and experimental detection of sRNAs

Prior to analyzing the sequencing reads, adaptors were removed and the Illumina pair-end reads were quality checked using FASTQC: Read QC and trimmed using Trim sequences (version 1.0.0) implemented within the Galaxy software [54–56]. Quality trimmed reads were mapped to the *P. atrosepticum* SCRI1043 genome (http://www.ncbi.nlm.nih.gov/nuccore/50118965?report=fasta) using Bowtie2 [57]. The mapped reads in SAM format were converted to sorted and indexed BAM files using SAMtools version 0.1.18 [19]. Each BAM file was split into two separate forward and reverse strand alignments using SAMtools to obtain transcriptional direction. For visualization of the data in a strand-specific manner, the genome browser Artemis [58], was used. The strand-specific RNA Sequencing data from this study have been deposited in NCBI’s Gene Expression Omnibus (GEO) with the accession number GSE68547.

### RT-PCR validation of novel sRNA candidates

For RT-PCR, first-strand cDNA was synthesized from 1 μg of total RNA using Superscript™ III First-Strand cDNA Synthesis SuperMix kit according to the manufacturer’s protocol (Invitrogen, USA). The first-strand cDNA samples were used for RT-PCR, which was performed on Bio-RAD T100^TM^ Thermal Cycler conventional PCR (Bio-RAD, USA). To check for genomic DNA contamination, a non reverse-transcriptase control was included. The sRNA primers were designed online using Primer3 (Additional file 8: Table S8). PCR was performed in a 25 μl reaction mix containing 1 μl of template cDNA (~40 ng), Taq DNA Polymerase, 10× Taq Buffer (New England Biolabs, UK), 2.5 mM dNTPs each and 0. 5 μM of forward and reverse primer each. Thermal cycling conditions were: 95 °C for 2 min; 30 cycles of 95 °C for 30 sec, 57 °C for 30 sec, 72 °C for 60 sec, and the final extension at 72 °C for 5 min. The PCR products were analysed on 1.5 % agarose gel including 100 bp DNA molecular weight ladder (NEB, UK).

### Differential expression analysis of sRNAs

Artemis genome browser was used to create features of the discovered sRNAs on the *P. atrosepticum* reference genome and to make read counts for reads aligning to each strand under each growth condition. The read counts were used as input for the sRNA differential expression analysis using edgeR [39]. sRNA transcripts were considered differentially expressed provided that the p-value was < 0.05 and q-value < 0.1.

### RT-qPCR validation of RNA-seq data

First strand cDNA synthesis was performed individually from total RNA samples from each of three biological replicates per condition using Superscript III First-Strand cDNA Synthesis SuperMix kit (Invitrogen, USA). For RT-qPCR, 2 μl of sample was added to 8 μl of Applied Biosystems SYBR Green Master Mix including each primer at a concentration of 0.4 μM and the reaction performed in the QuantStudio 12 K Flex Real-Time PCR system (Life Technologies, USA). The following cycling conditions were used: an initial denaturation at 50 °C for 5 min and 95 °C for 2 min, followed by 45 cycles of 95 °C for 15 s and 60 °C for 1 min. Each sample was run in triplicate. Relative expression was measured using the comparative 2-ΔΔ^ct^ method [59] after normalizing the samples to *recA* as the reference gene. Primers were designed using Primer3Plus (http://primer3plus.com/cgi-bin/dev/primer3plus.cgi) (Additional file 9: Table S9).

### Computational (*in silico*) sRNA prediction

#### Soft rot bacteria genome sequences

The genome sequences of six soft rot bacteria species (*Pectobacterium atrosepticum* SCRI1043, *P. carotovorum* subsp. *carotovorum* PC1, *P. wasabiae* WPP163, *Pectobacterium* sp. SCC3193, *D. dadantii* Ech703, and *Dickeya zeae* Ech1591 were obtained from the European Nucleotide Archive (http://www.ebi.ac.uk/ena/).

#### Identification of RITs

The WebGeSTer DB [60], database was used in this study to predict Rho-independent terminators (RITS) in *P. atrosepticum* SCRI1043 using default parameters. Briefly, no more than three mismatches were permitted within the stem structure and only RIT candidates with the highest ∆G score (∆G < = − 12.0 kcal/mol) were considered. Coordinates for putative RITs were obtained from the WebGeSTer DB, and java scripts were used to extract the sequences 200 nt upstream of the terminators (including the stem loop and tail sequences of the terminator). These sequences were considered as putative sRNA candidates and used in downstream sRNA prediction analysis. Additionally, known sRNAs within the *P. atrosepticum* SCRI1043 genome sequence were searched in the SIPHT web interface (published annotations) [28].

### sRNA conservation analysis

The conservation of sRNA sequences detected using deep sequencing was determined by similarity analysis against sequences of complete genomes of five soft rot Enterobacteriaceae species using YASS [29], a sequence similarity search tool, with standard parameters.

### Classification of sRNA

Following the model implemented for *Escherichia coli* [27], custom scripts written in java were used to classify the predicted sRNA candidates into five non-coding RNA groups based on their position in relation to adjacent CDSs. Briefly, the first nucleotide in each RIT was used as the representative position of each sRNA candidate. To determine asRNA, the reference nucleotide on the opposite DNA strand had to be at least +15 nt relative to the ATG codon to − 50 nt with respect to the stop codon. For 5′ UTR, sRNA candidates had to be on the same DNA strand as the CDS and in a distance of < −100 nt upstream the ATG codon and for 3′ UTR between +60 and +200 nt downstream of the stop codon. The rest of the remaining putative sRNAs were considered as IGR candidates if they were outside a CDS**.**

## Additional files

Additional file 1: Table S1. Complete list of RNA-seq detected sRNAs (XLSX 19 kb) Additional file 2: Table S2. Predicted transcription start sites of RNA-seq detected sRNAs (XLSX 9114 kb) Additional file 3: Table S3. List of *in silico* predicted sRNAs using RITs from WebGester DB. S2A: Forward strand predictions. S2B: Complementary strand predictions (XLSX 30 kb) Additional file 4: Table S4. Combined list of predicted sRNA using SIPHT and RITs from WebGester DB. S3A: Matches of *in silico* predictions with SIPHT (forward strand) S3B: Matches of in silico predictions with SIPHT (complementary strand) (XLSX 15 kb) Additional file 5: Table S5. Conservation analysis in Soft Rot *Enterobacteriaceae (XLSX 16 kb)*
Additional file 6: Table S6. Confirmation of RT-PCR amplicons by sequencing and BLASTn against respective sRNA sequences (XLSX 9 kb) Additional file 7: Table S7. Differentially expressed sRNA under nutrient-rich and starvation conditions (XLSX 19 kb) Additional file 8: Table S8. List of primers used for RT-PCR validation of novel sRNAs (DOCX 12 kb) Additional file 9: Table S9. List of primers used for RT-qPCR validation of RNA-seq expression data (DOCX 12 kb)

## Abbreviations

3′ UTR: 3′ untranslated regions
5′ UTR: 5′ untranslated regions
asRNAs: antisense RNAs
BLAST: basic local alignment search tool
CDSs: protein-coding regions
CFU: colony forming units
EPS: extracellular polysaccharides
IGR: intergenic regions
mRNA: messenger RNA
ORF: open reading frame
RITs: rho-independent terminators
RPKM: reads per kilobase of transcript per Million mapped reads
SRE: soft rot *enterobacteriaceae*
SRNA: small RNA
ssRNA-seq: strand-specific RNA sequencing
TPP: thiamine pyrophosphate
TSS: transcriptional start sites
