# Supplementary material for: Discovery and profiling of small RNAs responsive to stress conditions in the plant pathogen Pectobacterium atrosepticum
Source: BMC Genomics. 2016 Jan 12;17:47. doi: 10.1186/s12864-016-2376-0 (PMC4710047; doi:10.1186/s12864-016-2376-0)
Supplement: Additional file 8: Table S8. — List of primers used for RT-PCR validation of novel sRNAs (DOCX 12 kb) [file 12864_2016_2376_MOESM8_ESM.docx]

| **Primer name** | **Sequence 5' 🡪 3'** |
| --- | --- |
| fwd_6_F | AATTAAATAACAGTATTGTGTGACCTG |
| fwd_6_R | TTTTTCACGGGAACAAAGG |
| fwd_42_F | GAGTGGCCAAATGAGGTAGC |
| fwd_42_R | GCCTGAGTACACAAGTGCAAG |
| fwd_44_F | CGGGATGTCTCGTCAAAAAT |
| fwd_44_R | CAAAAATATGCCGCCTTCC |
| fwd_72_F | AAATGAACAATGCCTGTAGGG |
| fwd_72_R | TCGTTACAGCATTAACCTGCAT |
| rev_11_F | ATCGTGGTATCCAGCGGTTT |
| rev_11_R | GGTTAAGGATGCCACCTTTG |
| rev_13_F | TAATCATGAAGCCGTGAAGC |
| rev_13_R | CTTGATTCTCAACCCCGAAT |
| rev_24_F | ACTGCTGCTTGCAAATGATG |
| rev_24_R | AAGATCGCTATACGTCTTCGGTA |
| rev_39_F | CATGCCATGACTGACTGTGA |
| rev_39_R | GGGAATAACAGAATTTTATGCTGA |
| rev_41_F | GACAGCCTGAGATAATGAGCTG |
| rev_41_R | GCTCTGGCAAGACAGCTTCC |

Table S8: List of primers used for RT-PCR validation of novel sRNAs
