# Supplementary material for: Discovery and profiling of small RNAs responsive to stress conditions in the plant pathogen Pectobacterium atrosepticum
Source: BMC Genomics. 2016 Jan 12;17:47. doi: 10.1186/s12864-016-2376-0 (PMC4710047; doi:10.1186/s12864-016-2376-0)
Supplement: Additional file 9: Table S9. — List of primers used for RT-qPCR validation of RNA-seq expression data (DOCX 12 kb) [file 12864_2016_2376_MOESM9_ESM.docx]

| **Primer name** | **Sequence 5' 🡪 3'** |
| --- | --- |
| rprA_F | AAAAAGGCCCATCTCAGG |
| rprA_R | CGGAGCTAGTATTAACCTACTGTTTG |
| glmZ_F | ACGGGTAAGAAAGCATCACG |
| glmZ_R | AACAGGGCGTGAAAACGAAC |
| glmY_F | AACGTTATGTCCGGAAAGCC |
| glmY_R | ACGCAGAGCCAATTTTGAGG |
| ryhB_F | CACCCGAGCTGGCTAAATAATAC |
| ryhB_R | GTGTTTATGACGATCCGGCTTC |
| recA_F | TCGATGCTGAACATGCGTTG |
| recA_R | GCATCACAAATCTCCAACGC |
| rev_31_F | ACGTCGTTCCGATTTTGTGC |
| rev_31_R | TCCGATGTTTGCCGATAAACC |
| fwd_55_F | TCAGCATGTGCAGAAGTCTG |
| fwd_55_R | ATGCACTGGTGAGAAACTGC |
| fwd_20_F | AATCGTGCCGTTTTCTTCCG |
| fwd_20_R | TGCATTTCAATCGGCTGTGG |
| reg_seq70_F | TTTGTCAGCAATCGCAGCAC |
| reg_seq70_R | TTAATGAAGCGCTGCGTGAG |

Table S9: List of primers used for RT-qPCR validation of RNA-seq expression data
